# Metabolic Reprogramming in Sickle Cell Diseases: Pathophysiology and Drug Discovery Opportunities

**DOI:** 10.3390/ijms23137448

**Published:** 2022-07-04

**Authors:** Dina Alramadhani, Anfal S. Aljahdali, Osheiza Abdulmalik, B. Daniel Pierce, Martin K. Safo

**Affiliations:** 1 Department of Medicinal Chemistry and the Institute for Structural Biology, Drug Discovery and Development, School of Pharmacy, Virginia Commonwealth University, Richmond, VA 23298, USA; alramadhanid@vcu.edu; 2 Department of Pharmaceutical Chemistry, King Abdulaziz University, Alsulaymanyah, Jeddah 21589, Saudi Arabia; ashaljahdali@kau.edu.sa; 3 Division of Hematology, The Children’s Hospital of Philadelphia, Philadelphia, PA 19104, USA; abdulmalik@chop.edu; 4 Department of Biology, University of Richmond, Richmond, VA 23173, USA; bpierce@richmond.edu

**Keywords:** sickle cell disease, glycolysis, pentose phosphate pathway, Embden–Meyerhof–Parnas pathway, 2,3-biphosphoglycerate, pyruvate kinase, bisphosphoglycerate mutase, glyceraldehyde-3-phosphate dehydrogenase, triosephosphate isomerase, glucose-6-phosphate dehydrogenase

## Abstract

Sickle cell disease (SCD) is a genetic disorder that affects millions of individuals worldwide. Chronic anemia, hemolysis, and vasculopathy are associated with SCD, and their role has been well characterized. These symptoms stem from hemoglobin (Hb) polymerization, which is the primary event in the molecular pathogenesis of SCD and contributes to erythrocyte or red blood cell (RBC) sickling, stiffness, and vaso-occlusion. The disease is caused by a mutation at the sixth position of the β-globin gene, coding for sickle Hb (HbS) instead of normal adult Hb (HbA), which under hypoxic conditions polymerizes into rigid fibers to distort the shapes of the RBCs. Only a few therapies are available, with the universal effectiveness of recently approved therapies still being monitored. In this review, we first focus on how sickle RBCs have altered metabolism and then highlight how this understanding reveals potential targets involved in the pathogenesis of the disease, which can be leveraged to create novel therapeutics for SCD.

## 1. Introduction

### 1.1. Pathophysiology of Sickle Cell Disease

Sickle cell disease (SCD) is a genetic disorder that affects millions of people worldwide [1,2]. The pathophysiology of the disease is directly associated with a pathogenic mutation in the oxygen (O_2_) transport protein, hemoglobin (Hb) [2,3]. The primary function of Hb is to transport oxygen from the lungs to the tissues, binding and releasing the oxygen cooperatively, the latter with the help of the natural allosteric effector, 2,3-bisphosphoglycerate (2,3-BPG). During that process, Hb equilibrates between two conformational states: (1) the high O_2_-affinity relaxed (R) Hb, or oxygenated Hb, and (2) the low O_2_-affinity tense (T) Hb, or deoxygenated Hb (deoxyHb) [2,4]. Normal human adult Hb (HbA) is a tetrameric protein consisting of two α-subunits (α_1_ and α_2_) and two β-subunits (β_1_ and β_2_) in a tetrahedral arrangement with a large central water cavity. Each subunit has a prosthetic heme group that binds ligands, including oxygen. Several naturally occurring Hb variants have been implicated in SCD pathologies [4,5,6,7,8,9,10], with the most well-known variant sickle Hb (HbS), resulting from a single-nucleotide mutation in the β-globin gene that codes for βVal6 instead of βGlu6 [7,8]. Unlike HbA, HbS tends to polymerize or aggregate under hypoxia or when deoxygenated, because of an interaction between the pathological β_2_Val6 from one deoxyHbS molecule and a hydrophobic acceptor pocket in a proximate deoxyHbS molecule containing the amino acids β_1_Ala70, β_1_Phe85, and β_1_Leu88 [2,3,8]. The initial polymer formation becomes pathological only when stabilized by several secondary contacts between the HbS molecules. Consistently, mutations that break some of these secondary contacts, for example, αAsn78→Lys (Hb Stanleyville), βAsp73→Asn (Hb Korle Bu), or βAsp73→Val (Hb Mobile), increase the solubility of deoxyHbS, thereby reducing the severity of the disease [2,4,8,9,10]. Interestingly, 2,3-BPG, which is found in high concentration in sickle erythrocytes or red blood cells (RBCs), in conjunction with the hypoxia-responsive signaling molecule, sphingosine 1-phosphate (S1P) [11], adversely decreases HbS affinity for oxygen, leading to an increased concentration of the polymer-forming low-O_2_-affinity deoxyHbS [12,13,14,15,16,17,18]. Polymerization results in the formation of a helical structure, which ultimately leads to the sickling of RBCs and impaired rheology of the blood [2,3,7,8,10]. Adhesion between the sickled RBCs, neutrophils, endothelium, and platelets occurs, leading to vaso-occlusion (VOC) that eventually results in complications, such as ischemia, painful VOC crises, and acute chest syndrome [2,3,19,20,21]. Sickle RBCs are also susceptible to oxidative damage and hemolysis, resulting in hemolytic anemia [2,3,19,20,21]. Other complications of the disease include stroke, bone infarcts and necrosis of the femoral head, leg ulcers, splenic infarction, and pulmonary hypertension [2,3,19,20,21].

### 1.2. Current Treatments of SCD

Four drugs, namely voxelotor, Endari, crizanlizumab, and hydroxyurea, are currently approved for the treatment of SCD [2,3,22,23,24,25,26,27]. Voxelotor (Oxbryta), developed by Global Blood Therapeutics, increases Hb affinity for oxygen to prevent the hypoxia-induced polymerization and the ensuing sickling of RBC. Clinically, voxelotor increases Hb levels and reduces hemolysis in patients [3,24,26,27]. Although not conclusive, recent evidence also suggests that this drug may have the potential to reduce painful VOC crises [28]. Voxelotor’s therapeutic effect, however, may be limited in regions of severe hypoxia since it requires oxygen for its antisickling mechanism of action. Moreover, the effect of voxelotor increasing Hb oxygen affinity is inherently limited by the need to avoid impeding oxygen unloading to tissues. Endari (L-glutamine), developed by Emmaus Life Science, reduces complications of SCD associated with oxidative stress; however, the European regulatory body recommended against approval of the medication due to limited evidence of efficacy in Phase III trials [25]. Crizanlizumab (ADAKVEO), a monoclonal antibody developed by Novartis, reduces the frequency of VOC crises and complications of SCD by preventing P-selectin-mediated adhesion of RBCs to the vascular endothelium [23]. Finally, hydroxyurea (HU) has been used for over two decades to treat SCD [2,3,22,29]. It increases the production of fetal hemoglobin (HbF) to prevent polymerization of HbS [2,22,29]. For a variety of reasons, including poor compliance, the use of HU has remained limited globally [30,31]. Other available treatment options for treating SCD are transfusion therapy, hematopoietic cell transplantation [32,33], and gene therapy [34,35,36], and while the last two therapies are very promising, they are potentially limited by cost and general accessibility. None of the current therapeutics for SCD is likely to be universally effective or feasible, therefore the need remains for newer therapeutic options.

### 1.3. 2,3-BPG in SCD Pathogenesis and Potential Targets for SCD Therapeutics

Several studies have implicated 2,3-BPG in the pathogenesis of SCD [2,12,13,14,15,16,17,18]. The high level of 2,3-BPG in sickle RBCs has been shown to increase the hypoxia-induced HbS polymerization and subsequently RBC sickling [2,37]. In more recent studies, the Xia group has shown that, under hypoxic conditions, the sickle RBCs undergo metabolic reprograming induced by elevated levels of S1P as a result of increasing the activity of sphingosine kinase 1 (SphK1) [12,13,38]. The elevated level of S1P, in conjunction with increased levels of 2,3-BPG, results in switching glucose flux toward glycolysis or the Embden–Meyerhof–Parnas pathway (EMP) relative to the pentose phosphate pathway (PPP) that leads to disease progression [12]. These observations open a plethora of novel targets for new classes of therapeutics for SCD. 

The aim of this review is to provide a brief overview of glucose metabolism via the EMP and the PPP pathways, including how altered metabolism in both pathways in sickle RBCs may contribute to SCD pathogenesis. Further, we discuss how specific enzymes involved in both metabolic pathways could be targeted for drug development for SCD.

## 2. Glucose Metabolism

Red blood cells are primarily responsible for transporting oxygen from the lungs to the tissues and returning carbon dioxide from the tissues to the lungs as a byproduct; both processes require energy to operate properly [39]. As RBCs lack intracellular organelles, their primary source of energy is via anaerobic glycolysis [39,40,41]. Glucose molecules are broken by two important pathways—glycolysis, also referred to as the EMP pathway, and the PPP, also referred to as hexose monophosphate shunt (HMP) [41,42,43,44,45]. The EMP pathway, the main pathway in glucose metabolism, breaks down glucose into pyruvate or lactate, producing adenosine triphosphate (ATP) as a source of energy to accomplish cellular processes [41,42,45]. It also facilitates the production of the reducing agent, nicotinamide adenine dinucleotide (NADH), as well as 2,3-BPG to regulate the oxygen carrying capacity of Hb [41,42,45]. The HMP shunt, however, is an alternate pathway in which glucose is broken down into different metabolic intermediates required for cellular protection against oxidative damage [39,41,42,43,45]. The flux of glucose through both pathways depends on O_2_ variation in the cells [39,41,42,43]. When RBCs are oxygenated, glucose metabolism via EMP is inhibited, while metabolism through PPP is induced to combat the oxidant stress [39,41,42,43,45]. In contrast, when RBCs are deoxygenated, glucose metabolism through the EMP pathway is induced to compensate for the hypoxia [39,41,42,43,45]. The activities of the glycolytic enzymes are crucial to maintain RBC homeostasis, and dysfunction of any of these enzymes or the redox system can lead to several hematological disorders and/or their underlying pathophysiologies [46].

### 2.1. Embden–Meyerhof–Parnas Pathway (EMP)

**Figure 1:**
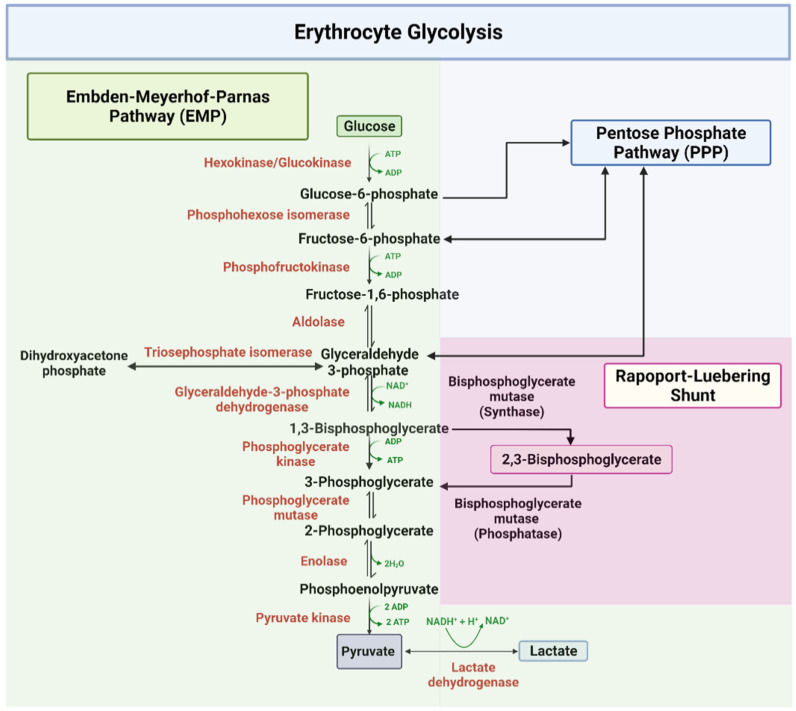
The glycolytic pathway [41,42,43,45].

The EMP or glycolysis pathway provides cellular and metabolic energy for biomass production (Figure 1). Detailed description of the ten enzyme-catalyzed reactions in the EMP pathway has been extensively documented in several review articles and books [41,42,43,45]. About 90% of glucose is metabolized through this pathway under normal physiological conditions. In all, a total of two net molecules of ATP and two NADH are produced per one molecule of glucose during glycolysis. As shown in Figure 1, glycolysis starts with the phosphorylation of glucose by the enzyme hexokinase, which utilizes ATP to produce glucose-6-phosphate (G-6-P), and ends in the phosphorylation of adenosine diphosphate (ADP) to ATP by the enzyme pyruvate kinase (PK) [41,42,43,45]. In addition 1,3-bisphosphoglycerate (1,3-BPG) produced by glyceraldehyde-3-phosphate dehydrogenase (GAPDH) and subsequently converted into 3-phosphoglycerate (3-PGA) to produce ATP by phosphoglycerate kinase (PGK) [41,42,43,45], may bypass the ATP producing step at PGK and shunt into a side glycolytic pathway known as the Rapoport–Luebering pathway, where it is converted by the synthase activity of bisphosphoglycerate mutase (BPGM) into 2,3-BPG [47]. BPGM also reversibly acts to hydrolyze 2,3-BPG to 3-PGA, which then re-enters the main glycolytic pathway. 2,3-BPG, as mentioned earlier, has a significant contribution to SCD pathogenesis due to its elevation in sickle RBCs [2,12,13,15,16,17]. Moving forward, BPGM and/or GAPDH could potentially serve as a target to modulate the concentration of 2,3-BPG in erythrocyte with a therapeutic effect on SCD. The last glycolytic enzyme, PK, may also be a target for SCD drug discovery as increasing its activity may lead to the removal of 2,3-BPG from the cell. 

### 2.2. The Pentose Phosphate Pathway (PPP)

**Figure 2:**
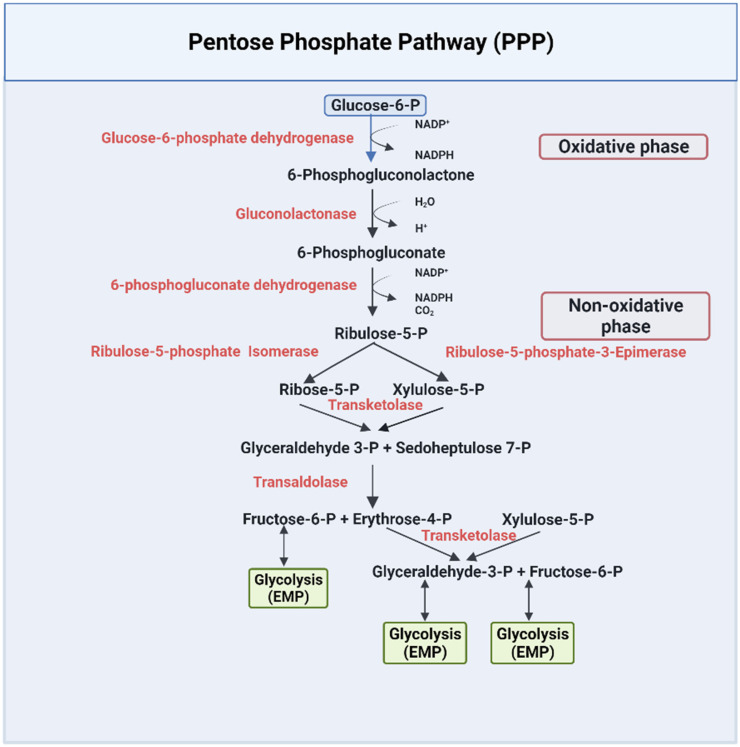
The pentose phosphate pathway [42,43,45,48].

The PPP pathway, or HMP, is a metabolic pathway that is parallel to the EMP pathway (Figure 2). Only 10% of glucose is metabolized through this pathway under normal physiological conditions [43]. The PPP has an irreversible oxidative phase, where reduced nicotinamide adenine dinucleotide phosphate NADPH is formed, and a reversible nonoxidative phase, where 5-carbon sugars are synthesized [43]. The PPP pathway has been extensively documented in several review articles and books [42,43,45,48]. Due to lack of mitochondria, erythrocytes solely depend on this pathway to combat oxidative damage by producing NADPH [43,48], which is utilized by several enzymes as a cofactor to reduce toxic radicals. For example, NADPH is a cofactor for glutathione reductase (GR), which is responsible for converting the oxidized state of glutathione (GSSG) to the reduced state of glutathione (GSH) [49]. GSH is used by multiple enzymes as a cofactor to prevent the destruction of cells, including RBCs, by reactive oxygen species [50,51]. NADPH also serves as a cofactor to the enzyme methemoglobin reductase for reducing methemoglobin to ferrous Hb, a crucial process for maintaining RBC structure and integrity [52].

## 3. Glucose Metabolism and Pathophysiology of SCD

### 3.1. SCD and the Glucose Flux Switch toward Glycolysis Relative to the Pentose Phosphate Pathway

From the forgoing, it is evident that the balance between the activities of the EMP and PPP pathways is crucial for the normal physiological function of erythrocytes. Metabolic reprogramming in sickle RBCs, where PPP glucose flux is switched toward glycolysis, is expected to cause an increase in 2,3-BPG production while decreasing the production of NADPH, and, subsequently, a decrease in the concentration of the antioxidant GSH [12,13,53,54,55,56]. This metabolic switch will impact the ability of sickle RBCs to detoxify the reactive oxygen species, leading to a cascade of events that ultimately worsens the symptoms of SCD [12,13,38,55,56].

Thus, targeting the PPP pathway, especially to increase the production of NADPH, may serve to decrease oxidative stress in sickle RBCs and provide a therapeutic option for SCD. Interestingly, one of the recently approved drugs, Endari (L-glutamine) works by reducing oxidative stress and the associated complications of the disease [25]. During the oxidative phase of the PPP pathway, two NADPH are produced by two-step catalyzed reactions by the enzymes glucose-6-phosphate dehydrogenase (G6PDH) and 6-phosphogluconate dehydrogenase (6PGDH) (Figure 2) [57]. These enzymes are, therefore, potential targets for increasing the production of NADPH to counter the serious pathological problem of oxidative stress in sickle RBCs.

### 3.2. The Role of 2,3-BPG in SCD Pathophysiology

The oxygen carrying function of Hb is closely associated with 2,3-BPG. Under normal physiological conditions, only about 25–40% of the oxygen bound to Hb is released to tissue, which is made possible by the preferential binding of 2,3-BPG at the β-cleft of deoxyHb to decrease the protein affinity for the bound oxygen [4,58]. In sickle RBCs, the concentration of 2,3-BPG is significantly elevated, which allows rapid and increased release of oxygen even before the blood reaches the tissue beds [14]. This adaptive response is required to counter the chronic anemia due to the loss of Hb from constant hemolysis by the brittle sickle RBCs [4,14,59,60]. Nevertheless, this response is counterproductive since it leads to an increased concentration of the polymer-forming deoxyHbS. Evidence also suggests that 2,3-BPG is involved in direct stabilization of the HbS polymers [37]. Unlike individuals carrying the homozygous sickle cell gene (HbSS), who suffer from severe illness, individuals with sickle cell trait (HbAS) usually exhibit no significant clinical symptoms. Interestingly, HbAS individuals with inherited PK deficiency have the same severe clinical phenotypes as HbSS individuals due to high elevation of 2,3-BPG concentration in the RBCs, further supporting the importance of 2,3-BPG in disease pathogenesis [61,62]. Expectedly, decreasing 2,3-BPG levels in sickle RBCs has been shown to reduce HbS polymerization and RBC sickling [16,17].

### 3.3. The Combinatorial Role of 2,3-BPG and S1P in SCD Pathophysiology

Sphingosine-1-phosphate (S1P) is a signaling molecule involved in regulating many cellular processes, such as angiogenesis, cell proliferation, migration, endothelial injury, and inflammation [11]. S1P has been shown to be elevated in the blood of humans and mice with SCD due to the increased activity of sphingosine kinase 1 (SphK1) promoting sickling, hemolysis, inflammation, and multiple tissue damage [12,13]. Sphk1 knockdown in SCD mice significantly reduced sickling due to lowering S1P levels in erythrocytes [12]. Moreover, genetic deletion of Sphk1 in SCD mice was also observed to significantly lower 2,3-BPG production [12]. Interestingly, 2,3-BPG and S1P work together synergistically to decrease Hb affinity for oxygen, promoting deoxygenation and contributing to erythrocyte sickling [12,13]. Under normal O_2_ tension, the main glycolytic enzymes, such as GAPDH, aldolase, phosphofructokinase, pyruvate kinase, and lactate dehydrogenase form a complex with the RBCs membrane protein Band 3 (cdB3), rendering the enzymes inactive [12,13]. However, in low O_2_ tension, deoxyHb binds to cdB3 (mediated by S1P and 2,3-BPG), to displace and release the glycolytic enzymes from cdB3 into the cytosol [12,13]. This leads to activation of glycolysis, which promotes glucose to enter the glycolytic pathway rather than the PPP, ultimately leading to a suppression of glutathione production and a subsequent increase in oxidative stress [12,13]. The enhanced glycolysis also increases production of 2,3-BPG, resulting in increased formation of the polymer-forming deoxyHbS, and the associated HbS polymerization and RBC sickling [12,13]. Interestingly, S1P only binds to deoxyHb in the presence of 2,3-BPG, which again serves to highlight the crucial role of 2,3-BPG in SCD pathogenesis [12,13].

## 4. Drug Discovery Opportunities and Challenges

From the previous discussion, proper balance between the PPP and EMP pathways is crucial for maintaining RBC health. Disfunction of any of the enzymes in these pathways and/or the redox system can lead to several hematological disorders, e.g., hemolytic anemia, and/or contribute to disease progression, e.g., SCD. In this section, we discuss some of the potential targets present in the two pathways that can be leveraged for novel therapeutics for SCD and the challenges of such efforts. 

### 4.1. Glycolytic Enzymes

#### 4.1.1. Erythrocyte Pyruvate Kinase (PKR)

**Figure 3:**
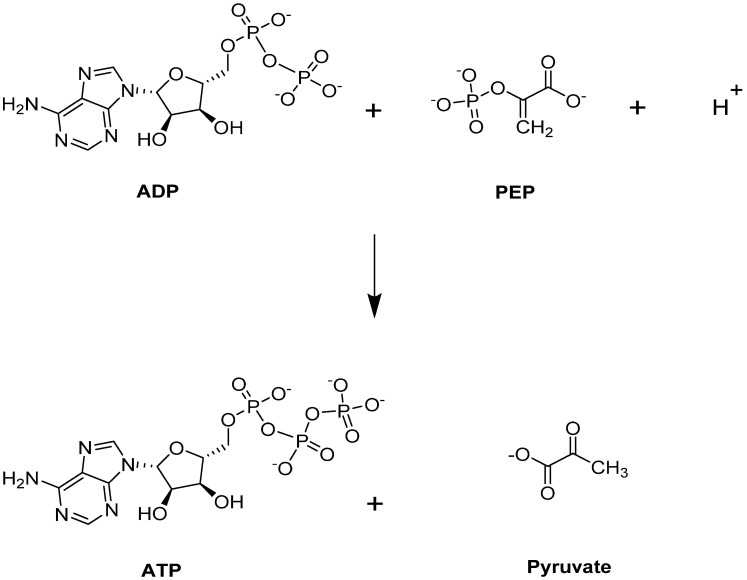
PKR-catalyzed reaction.

Pyruvate kinase (PK) catalyzes the last step of glycolysis, by transferring one phosphate group from PEP to generate the final product, pyruvate, with simultaneous production of ATP (Figure 3) [41,42,45,63]). Besides its typical role providing an energy source for the cell, ATP is a critical intermediate metabolite for maintaining the integrity and flexibility of the RBC membrane [40]. PK is responsible for producing 50% of ATP during glycolysis [64], and targeting this step could potentially benefit those with SCD. More importantly, PK activation would lead to a depletion of 2,3-BPG, a major contributor to SCD pathophysiology as it is downstream from the Luebering–Rapoport shunt. 

PK has four different isoenzymes in human tissue: (1) PKL, which is mainly found in the liver; (2) PKR, which is found in the RBCs; (3) PKM1, which is found in the muscles, heart, and brain; and (4) PKM2, which is found in early fetal tissue [65,66]. PKL and PKR isoenzymes are expressed from the gene *PKLR*, while PKM1 and PKM2 are expressed from the gene *PKM*. 

PKR-deficient reticulocytes, caused by a mutation in the PKLR gene, have been shown to have a reduced lifespan through selective destruction in the spleen [67]. Additionally, ATP depletion in PKR-deficient reticulocytes cells leads to increased RBC dehydration and destruction [68,69], causing chronic nonspherocytic hemolytic anemia (CNSHA) [64]. For this reason, PKR activators have potential application for the treatment of hemolytic anemia caused by pyruvate kinase deficiency. PKR deficiency is also associated with reduced Hb oxygen affinity as a result of increased 2,3-BPG production, which leads to an increased concentration of the polymer-forming deoxyHbS [62]. The involvement of PKR activity in the regulation of ATP and 2,3-BPG makes it a potential target for SCD therapeutics. In fact, two PKR activators, AG-348 and FT-4202, are currently in clinical trials for the treatment of SCD [70,71].

**Figure 4:**
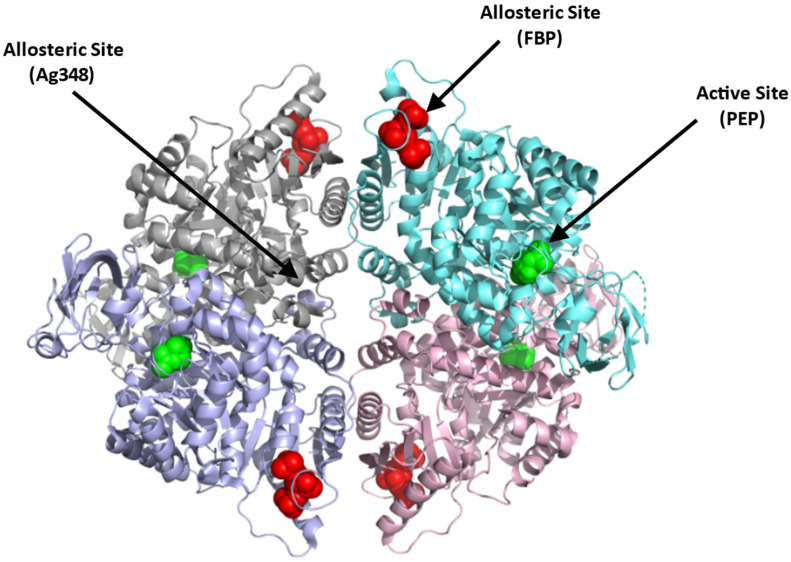
Crystal structure of PKR (PDB:2VGB) showing the allosteric site with FBP bound (red sphere), dimer–dimer interface binding site where the synthetic AG-348 is known to bind, and the active site with bound PEP (green sphere). The four subunits are shown in ribbons and colored gray, cyan, purple, and pink.

Structurally, PKR exists as a homotetramer, with each monomer composed of three domains A, B, and C with a molecular weight of 62 kDa per monomer (Figure 4; [66]) (PDB: 2VGB). PKR has three known binding sites that can be potentially targeted for therapeutics. These include the active site, the allosteric site, and the AG-348 binding site (Figure 4). The active site is located in a cleft between domains A and B, at the end of the (α/β)_8_ barrel and binds the substrates PEP and ADP. The allosteric site binds the natural activator, FBP, which causes a conformational change and an increase in the protein activity (Figure 4; [65,66]). Crystal structures of PKR variants revealed a conformational toggle between the open and closed positions of the allosteric loop, where in the absence of FBP, the open position is stabilized by a cation–π bond between Trp527 and Arg538′ (from an adjacent monomer) [72]. Interestingly, in some variants, glutamate is able to bind in place of FBP, leading to a partial allosteric activation [72]. Finally, a third binding cleft has been discovered to bind the RPK activator AG-348 (developed by Agios Pharmaceuticals). This site is deeply buried at the dimer–dimer interface suggesting a post-binding conformational change to PKR (Figure 4; [70]).

In contrast to the substrate binding (active) and the FBP binding (allosteric) sites that are highly charged, the AG-348 binding site is formed mostly by hydrophobic residues and few polar/basic residues [70]. Targeting the allosteric FBP binding site to activate the enzyme, thus poses a problem, since it may require synthetic ligands with charge moieties as found with FBP. Such compounds, even if potent, may not be bioavailable. The AG-348 binding site, and perhaps other potential binding cavities, thus serve as best target points to develop PKR activators. 

#### 4.1.2. Bisphosphoglycerate Mutase (BPGM)

**Figure 5:**
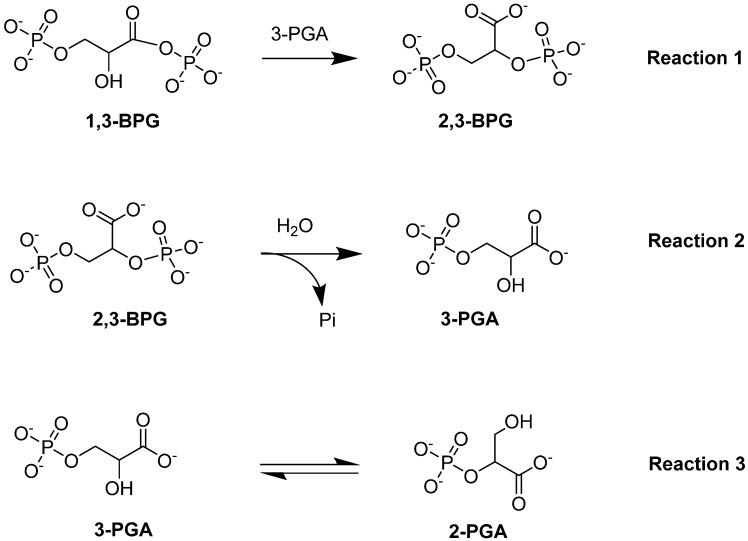
BPGM-catalyzed reactions: synthase activity (Reaction 1), phosphatase activity (Reaction 2), and mutase activity (Reaction 3).

Bisphosphoglycerate mutase (BPGM) is the central enzyme in the Rapoport–Leubering pathway, exclusively expressed in erythrocytes and placental cells [73,74]. BPGM regulates the intraerythrocytic level of 2,3-BPG by catalyzing both its synthesis and degradation [47,75]. The main activity of the enzyme is its synthase activity, which catalyzes the generation of 2,3-BPG from 1,3-BPG, an intermediate in glycolysis (Reaction 1; Figure 5). Alternatively, the phosphatase activity of BPGM leads to the hydrolysis of 2,3-BPG into 3-PGA and inorganic phosphate (Reaction 2; Figure 5). In addition to these two downstream effects, BPGM can function as a mutase, similar to the activity seen in the glycolytic enzyme phosphoglycerate synthase [76]. The mutase activity of BPGM involves catalyzing the interconversion between 2-PGA and 3-PGA in glycolysis (Reaction 3; Figure 5; [76,77]). This varying BPGM activities highlights its importance throughout these processes.

**Figure 6:**
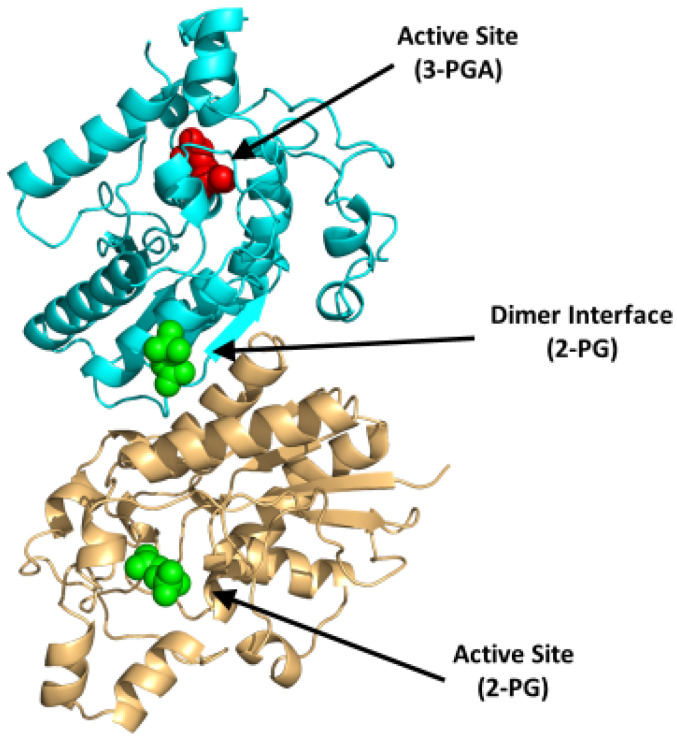
Crystal structure of BPGM (PDB:7n3r), showing the active site with bound 3-PGA (red sphere) or 2-PG (green sphere) and the dimer interface with bound 2-PG (green sphere). The two subunits are shown in ribbons and colored cyan and gold.

BPGM is a homodimer, with each monomer composed of two domains that are formed by six β-strands (named βA-F) and ten α-helices (named α1-10) with a molecular weight of 30 kDa per monomer [75,78]. The dimer is formed between the surface of the βC strands and α3 helices of the two monomers (PDB: 7n3r) (Figure 6). Interestingly, the three distinct reactions of BPGM are catalyzed at the same active site and share the same substrates and cofactors, imposing difficulties in assaying for 2,3-BPG production or BPGM activity. The phosphatase activity of BPGM has been reported to be activated by different effectors, such as chloride, sulfite, inorganic phosphate, and, most potently, 2-phosphoglycolate (2-PG) [77,79]. 2-PG is a physiological activator that exists in RBCs at a concentration of 2–5 µM [77]. A study published by Poillon et al. showed that activation of the phosphatase activity in the presence of glycolate resulted in a decrease of its 2,3-BPG level, consequently improving the solubility and ameliorating the sickling tendency of sickled RBCs [16,17]. Furthermore, a study by Knee et al. evaluated the effect of 2,3-BPG elimination with respect to the SCD pathology through a complete knockout of the BPGM gene in Townes model mice [80]. The BPGM-knockout mice had an increased Hb affinity for oxygen with a 59% reduction in RBC sickling [80]. While there are no known synthetic modulators of BPGM, its uniqueness to erythrocytes and its central role in 2,3-BPG production positions BPGM as an ideal target for SCD therapeutics. 

The Gly14 residue located at the bottom of the BPGM active site has been shown to play a role in substrate binding, as well as to modulate the three activities of BPGM differently (see Figure 5; [81]). Replacement of Gly14 to Ser led to a twofold increase and decrease in the mutase and phosphatase activities respectively, while keeping the synthase activity unchanged. On the other hand, replacing Gly14 with Arg enhanced phosphatase activity by about 29-fold, whereas the synthase and mutase activities diminished by 10-fold [81]. This study suggests the possibility of gene-based therapy as a way to treat SCD [81].

Although BPGM represents a potential target, there are no known lead compounds at present. Drug discovery requires reliable high-throughput assays, which currently are lacking for BPGM, partly because this enzyme is involved in three catalytic activities, including synthesis and degradation of 2,3-BPG at the same catalytic site. Additionally, the active site of BPGM is highly charged, thus posing a problem for designing synthetic modulators that are equally potent and bioavailable. In a recent published study, our group identified a novel binding site at the dimer interface of BPGM, which when bound with ligand appears to affect the catalytic activity of the enzyme [75]. This new site could be targeted for designing BPGM modulators, either to activate the phosphatase activity or to inhibit the synthase activity to reduce 2,3-BPG levels in RBCs. 

#### 4.1.3. Glyceraldehyde-3-phosphate Dehydrogenase (GAPDH)

**Figure 7:**
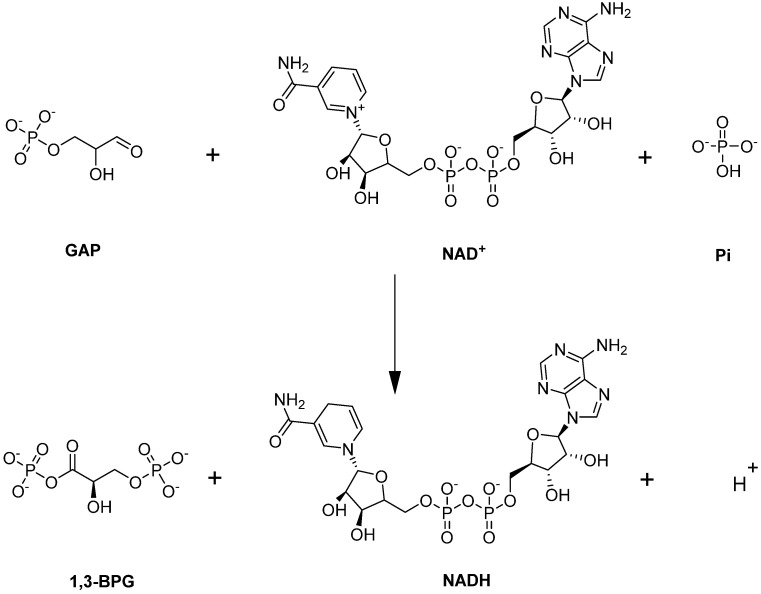
GAPDH-catalyzed reaction.

Glyceraldehyde-3-phosphate dehydrogenase (GAPDH) is a unique enzyme in glycolysis because it is located at the juncture of the EMP/PPP pathways. GAPDH catalyzes the sixth step of glycolysis—namely the conversion of GAP to 1,3-BPG with the simultaneous production of NADH (Figure 7; [82]).

**Figure 8:**
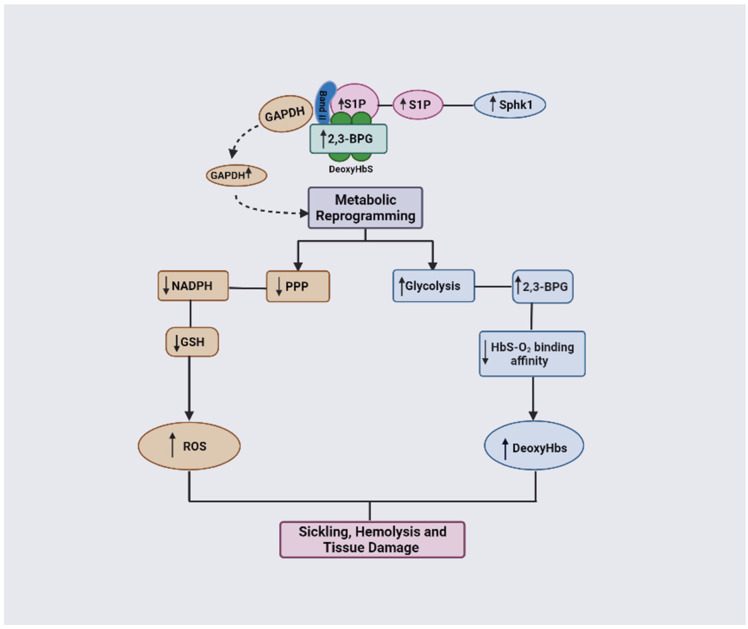
DeoxyHbS anchoring to membrane results in the release of GAPDH. Increased cytosolic GAPDH accelerates glycolysis and 2,3-BPG production while decreasing PPP and antioxidant production. Increased 2,3-BPG leads to increased formation of the polymer-forming deoxyHbS while a decreased antioxidant level causes more oxidative stress (ROS).

GAPDH functions by mediating O_2_-dependent metabolic variation through its facilitation of the binding of metabolic enzymes and deoxyHb to the N-terminal cytosolic domain of band 3 [12]. Normally, GAPDH is inactivated when bound to band 3 but becomes activated once it is released to the cytosol [12]. Under the RBC sickling condition, deoxyHbS forms a ternary complex with 2,3-BPG and S1P and binds with a high affinity to band 3, thereby displacing GAPDH into the cytosol, leading to an increased activity and consequent metabolic shift from PPP to glycolysis (Figure 8; [12]). Unfortunately, the increase in glycolysis results in a detrimental cycle in which 2,3-BPG levels are elevated, leading to an increased concentration of deoxyHbS and, thus, more RBC sickling. In turn, this causes a decrease in antioxidant production, making the cells unable to detoxify the reactive oxygen species and, ultimately, leading to RBC hemolysis and oxidative stress. It is worth noting that GAPDH is naturally inhibited by S-glutathionylation, which is severely reduced in SCD [83,84]. Therefore, the inhibition of GAPDH activity could potentially interrupt this cycle, as a therapeutic strategy to ameliorate SCD. 

**Figure 9:**
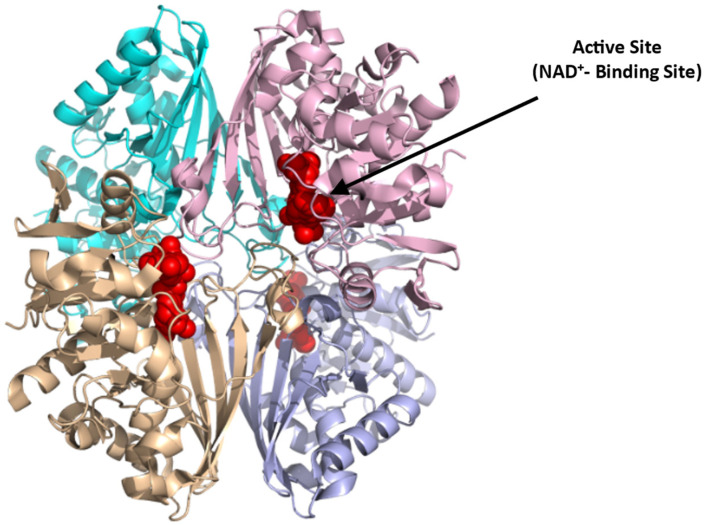
Crystal structure of GAPDH (PDB:1U8F), showing the active NAD⁺-binding site (red sphere). The four subunits are shown in ribbons and colored cyan, pink, brown, and purple.

GAPDH is a tetrameric protein (Figure 9; [85]),(PDB: 1U8F) composed of four identical monomers of 37 kDa. Each subunit consists of an NAD^+^-binding domain (residues 1–151, 315–335) and a catalytic domain (residues 152–314). The active site in each subunit resides in a large cleft between the NAD^+^-binding and catalytic domains. The nucleophilic cysteine (Cys152) resides at the N-terminus of the first helix of the catalytic domain [85]. The literature reports several GAPDH inhibitors, that presumably bind at the NAD^+^-binding site, for potential treatment of various diseases, including parasitic infections and cancers [86]. Examples are adenosine analogs that target trypanosomatid GAPDH for use to treat sleeping sickness [87] and koningic acid and DC-5163 which inhibit cancer cell proliferation by reducing ATP production [86,88]. GAPDH could also be targeted for SCD therapeutics since its inhibition can potentially reverse the metabolic switch back to PPP, relative to glycolysis in sickle RBCs, decreasing 2,3-BPG formation and oxidative stress. Potentially mitigating this positive effect is the apparent reduction in ATP production with GAPDH inhibition, which could pose a detrimental effect on the integrity and flexibility of the RBC membrane [40]. Moreover, because of the cellular abundance and ubiquitous nature of GAPDH [89], targeting this enzyme with small molecules could lead to potential systemic toxicity. Hence, for the drug to be potentially therapeutic, it should be selective and specific.

#### 4.1.4. Triosephosphate Isomerase (TPI)

**Figure 10:**
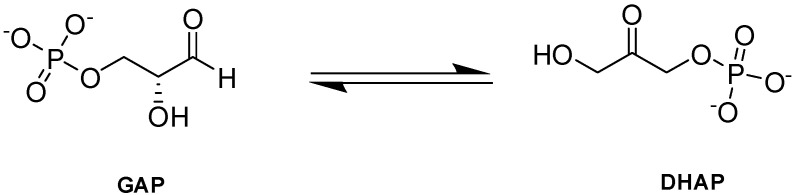
TPI-catalyzed reaction.

Triosephosphate isomerase (TPI or TIM) is a key enzyme in the glycolytic pathway that adjusts the equilibrium between GAP and DHAP, produced by aldolase in glycolysis (Figure 10; [90]).

GAP is central to both glycolysis and PPP, and it has been postulated that the PPP shunt works at near-maximal levels during TPI deficiency because PPP is linked to glycolysis through the intermediates G6P and GAP [91]. DHAP, alternatively, is a dead-end product in erythrocytes but is shunted into lipid metabolism in the brain [90]. DHAP is also known to decompose nonenzymatically to produce methylglyoxal that can lead to formation of glycation products (AGEs) [90,92]. Impairment or inactivation of TPI results in increased concentrations of PPP metabolites, as glucose is redirected to the PPP, thus increasing the formation of NADPH which helps to protect the cell from oxidative stress [93]. In fact, PPP activation has been proposed as a compensatory strategy for lower TPI activity [90]. Consistently, individuals with TPI deficiency showed an activation of G6PD, which catalyzes the rate-limiting phase of the PPP, further supporting this theory [90]. As such, TPI inhibitors could potentially be explored as possible SCD therapeutic agents. 

**Figure 11:**
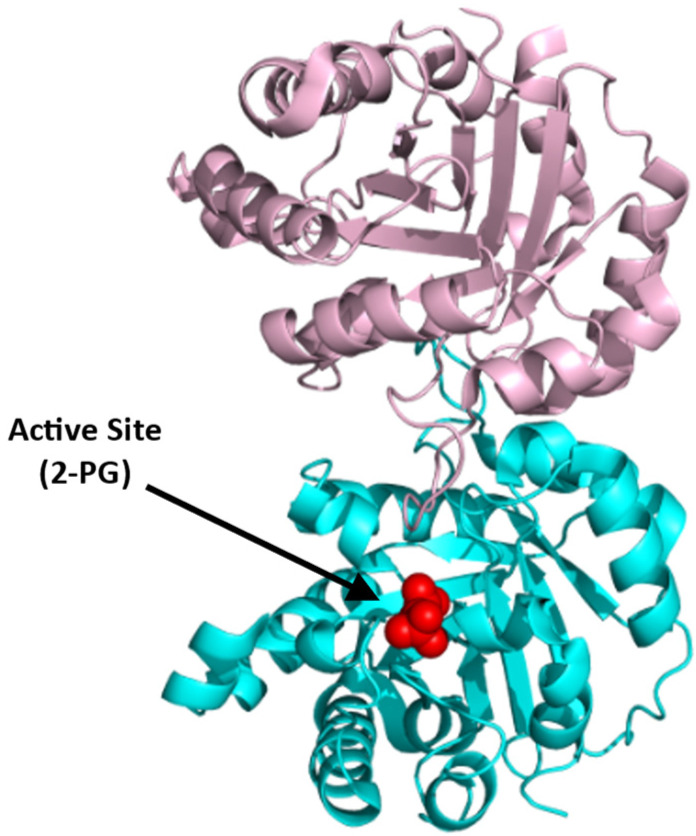
Crystal structure of TPI (PDB:1HTI), showing the active site with bound 2-PG (red sphere). The two subunits are shown in ribbons and colored pink and cyan.

Structurally, TPI is active as a homodimer. Each monomer consists of 248 amino acids with a molecular weight of 27 kDa (Figure 11; [94]), (PDB: 1HTI). The active site, which is composed of three conserved key residues, Lys13, His95, and Glu165, has been explored as a possible target for cancer therapy [95]. In addition to the active site, the nonconserved dimerization site of the parasitic TPI has been targeted by small-molecule inhibitors [96]. Both the active and dimerization sites in the human TPI could, therefore, be potentially targeted to develop SCD therapeutics. Since TIM is considered essential for energy production, inhibition may reduce ATP production, leading to a detrimental effect on the already fragile sickle RBC.

### 4.2. PPP Enzymes

#### Glucose-6-phosphate Dehydrogenase (G6PD)

**Figure 12:**
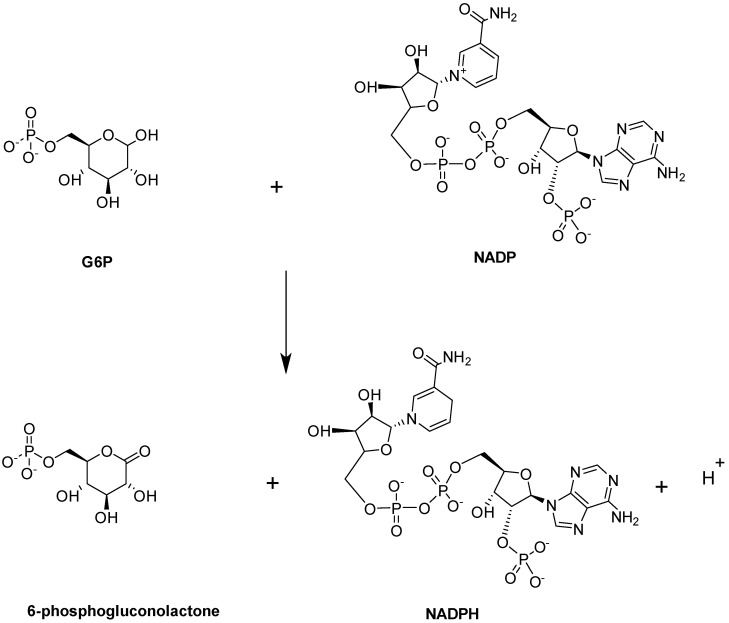
G6PD-catalyzed reaction.

Glucose-6-phosphate dehydrogenase (G6PD) enzymatic activity is the first and rate-limiting step in the PPP. It is expressed in most tissues and considered a ‘housekeeping’ enzyme. G6PD catalyzes the conversion of G6P into 6-phosphogluconolactne with the concomitant production of NADPH (Figure 12; [97]).

The main role of NADPH, which is a cofactor of G6PD, is to protect the cell from oxidative damage. Deficiency of G6PD, therefore, perturbs NADPH homeostasis, thereby impairing the ability of the cell to detoxify free radicals, leading to several pathological problems, such as hemolytic anemia [97]. G6PD deficiency has been linked to increased severity of anemia in SCD patients [98,99]. This, together with the fact that G6PD is the rate limiting step of PPP, suggests that activation of G6PD could be a potential strategy for managing SCD. 

**Figure 13:**
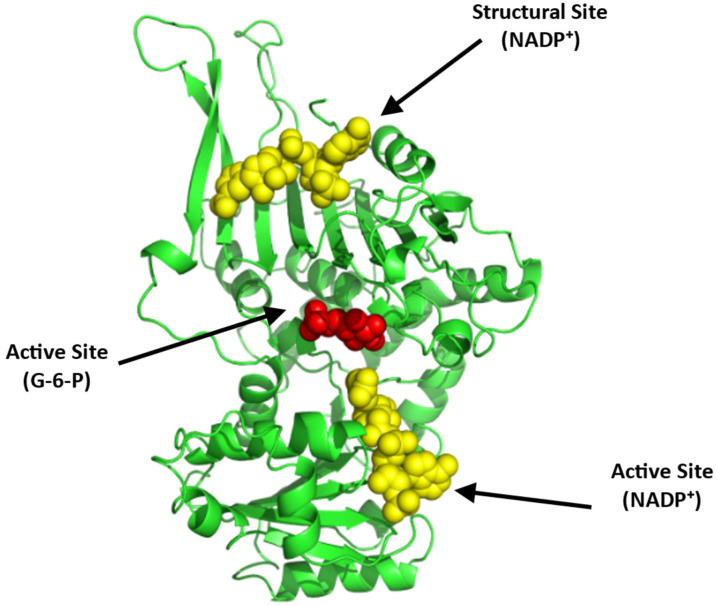
Crystal structure of G6PD (monomer), showing the active site with bound G-6-P (red sphere) and NADP⁺ (yellow sphere) and a structural site, also with bound NADP⁺ (yellow sphere). The two G6PD crystal structures (PDB: 2BHL and 2BH9) were superimposed using PyMOL to illustrate all the possible binding sites of G6PD. The subunit is shown in ribbons and colored green.

Structurally, G6PD equilibrates between a dimer and a tetramer with a molecular weight of 59 kDa per monomer (Figure 13; [100,101,102]). Each monomer consists of two co-enzyme (NADP^+^)-binding domains and a G6P-binding site that is located between these two domains (Figure 13). Recently, a small-molecule activator of G6PD, AG1, was discovered [101] and was suggested to bridge the dimer interface at the NADP^+^-binding sites of the two interacting G6PD monomers to induce a conformational change that activates its enzymatic function. While AG1 has not been tested for its effect on SCD, it was shown to reduce oxidative stress in zebrafish, which further supports the potential of G6PD activators as a class of future SCD therapeutics [101,102].

## 5. Conclusions

Sickle cell disease is an inherited chronic blood disorder that presents at birth. SCD is characterized by polymerization of deoxyHbS and concomitant sickling of RBCs [2,3,7,8,10]. Mature RBCs are responsible for transporting oxygen from the lung to tissues and they procure their energy via anaerobic glycolysis, which is required to maintain the structural integrity of the RBCs [4,40,41]. Two essential mechanisms break down glucose molecules: glycolysis or the EMP pathway, and the PPP or HMP pathway [41,42,43]. The flow of glucose across both channels is influenced by the amount of oxygen in the cells. Glucose metabolism via EMP slows when RBCs are oxygenated but speeds up via PPP to resist oxidant stress. When RBCs are deoxygenated, glucose is mostly metabolized via the EMP route to compensate for the hypoxia. Thus, maintaining RBC homeostasis requires the activity of several glycolytic enzymes. Hematological disorders and/or their associated pathophysiologies can be caused by a malfunction of any of these glycolytic enzymes and/or the redox system [41,42,46,52,53,54,55,103,104]. There are several lines of evidence to suggest that activation or inhibition of some of the glycolytic and PPP enzymes can lead to a reduction in crises of SCD. For example, activation of a subset of these enzymes, such as PK, BPGM, and G6PD, has been shown to have positive effects in SCD. Theoretically, inhibition of GAPDH and TPI could also help in the treatment of SCD. There are, however, several challenges posed by targeting the glucose metabolic pathway for drug discovery. Flux through the metabolic pathways is modulated through a combination of regulation of enzyme activity by small molecules, as well as regulation of protein levels by hormonal control of tissue-specific gene expression. Thus, targeting a specific enzyme, either totally inhibiting it or allosterically regulating it, could lead to modulation of the whole metabolic pathway as an adaptation mechanism, resulting in unintended consequences. Other challenges, specific to targeting some of the enzymes have also been discussed above. In summary, we have discussed glucose metabolism and how alterations in the two metabolic pathways, glycolysis and PPP, contribute to SCD pathogenesis. We have also highlighted how the enzymes participating in both pathways provide interesting SCD treatment targets for future research.

## Data Availability

Not applicable.
